# Ethnic Disparities in Health: The Public's Role in Working for Equality

**DOI:** 10.1371/journal.pmed.0030405

**Published:** 2006-10-24

**Authors:** David Satcher

## Abstract

If we had eliminated health disparities in the last century, then in the year 2000 alone there would have been 83,500 fewer African American deaths, says the 16th US Surgeon General.

As Assistant Secretary for Health (1998–2001) and Surgeon General (1998–2002) of the United States, I had the opportunity to lead in the development of Healthy People 2010. Healthy People 2010 is the current phase of the Healthy People Initiative, which began in 1979 with the vision of making America's communities healthier and happier places (http://www.healthypeople.gov). Healthy People 2010 is a comprehensive set of health objectives to be achieved over the first decade of the century. The overarching goals are to increase the quality of life and years of healthy life for all Americans and to eliminate racial and ethnic health disparities [1]. This has been an ambitious undertaking, but we are making progress.

Americans have gained 30 years in life expectancy in the last century overall, from 47 years in 1900 to 77.6 years in 2003. But African American men, with a life expectancy of 69.2 years compared to 76.1 years for African American women and 75.4 years for white men, continue to lag behind. Nevertheless, all groups have made significant progress in years of life lived. However, 50 percent of persons over 80 are incapacitated physically, mentally, or both—illustrating the importance of both quality of life and years of healthy life.

The significant gender and racial gaps for African American men are likely to persist because of death rates associated with the prevalence of cardiovascular disease, diabetes, hypertension, cancer, HIV/AIDS, and homicide among this group. African American men are more likely to die from these diseases, or their associated problems, and from homicide than their white counterparts.

The significant gender and racial gaps for African American men are likely to persist.

However, we have made significant progress in addressing racial and ethnic disparities in the number of children immunized and in screenings for breast cancer. But ethnic and racial disparities persist in areas such as diabetes, HIV/ AIDS, cardiovascular disease, and others for both African American men and women.

## What If We Were Equal?

In an attempt to put health disparities in perspective, for a recent special issue of *Health Affairs* devoted to racial and ethnic disparities, we asked the question, “What if we had eliminated disparities in health in the last century?” [2] By our calculations, there would have been 83,500 fewer black deaths overall in the year 2000 alone. That would have included about 24,000 fewer black deaths from cardiovascular disease.

If infant mortality had been equal across racial and ethnic groups in 2000, 4,700 fewer black infants would have died in their first year of life. Without disparities, there would have been 22,000 fewer black deaths from diabetes and almost 2,000 fewer black women would have died from breast cancer. Indeed, 250,000 fewer blacks would have been infected with HIV/ AIDS and 7,000 fewer blacks would have died from AIDS in 2000. As many as 2.5 million additional blacks, including 650,000 children, would have had health insurance in that year.

## Seeking Solutions

There are certain key determinants of health and of disparities in health. These include physical and social environments, individual behavior and biology, access to quality health care, and policies and interventions that affect people's health. Access to quality health care is a major barrier to successful health outcomes in the United States. African Americans and other racial and ethnic minorities are disproportionately affected by problems with access to quality care.

In seeking solutions to the problem of ethnic disparities, we all must be proactive as advocates for change. The general public can work to improve access to quality care by advocating for universal access to such care. This means we must participate in the democratic process and elect representatives who will support legislation that ensures the availability of and access to quality care for all.

Underrepresented minorities make up almost 30 percent of America's population, but they make up only 14 percent of the physicians in America [3]. This underrepresentation is a major barrier for minorities in accessing quality care. Since African American health professionals are five times more likely than majority professionals to serve African American patients, and Hispanic health professionals are three times more likely to serve Hispanic patients, we must work to elevate the representation of minorities in the health professions [4]. These groups should be introduced to the health professions at a young age and encouraged to pursue careers in these areas.

We must understand the relevance of culture to improving the quality of care. The cultural background of health-care providers influences how they interact with patients, and how they diagnose and treat health problems. We should also keep in mind that the cultural background of patients influences how, when, and where they present with illness and how they express it [5]. A culturally competent health-care provider or team is able to identify with, relate to, and accommodate the cultural background of the patient.

Lifestyle is another major determinant of health and is critical to the elimination of health disparities. The most important lifestyle indicators, according to the Leading Health Indicators of Healthy People 2010 (http://www.healthypeople.gov/LHI) and the Surgeon General's Prescription developed in 1999 (http://www.mediarelations.k-state.edu/WEB/News/NewsReleases/satchertext92001.html), are shown in Box 1.

While all of these factors are important and even crucial to eliminating disparities in health, the epidemic of overweight and obesity and its disproportionate impact on African Americans and other minorities is an increasing and troublesome problem in this country. Obesity is a major risk factor for cardiovascular disease, including hypertension and strokes, as well as for diabetes and cancer of the breast, colon, and prostate.

Communities must address policies at all levels of government to ensure support for education, physical exercise, and good nutrition in our schools. Some states have passed legislation in this regard.

Environment—both social and physical—is another major determinant of health disparities. The social environment must be targeted, especially environments of hopelessness that lead children to devalue themselves and to succumb to drugs, violence, and premature sexual activity. And we can all work to create safe and clean environments for children to grow up in.

**Box 1. Leading Health Indicators**

- physical activity
- good nutrition (especially consumption of fruits and vegetables)
- overweight and obesity
- avoidance of toxins, especially tobacco
- responsible sexual behavior, including delaying or abstaining from sex where appropriate and minimizing unplanned pregnancy and sexually transmitted diseases when sexually active

Finally, we must support research to gain a better understanding of disparities and to inform different approaches to eliminate them. Likewise, more African Americans need to participate in clinical trials for the same reasons. A recent study found that racial and ethnic minority groups in the US are as willing as non-minority individuals to participate in health research, but are underrepresented among the invited participants [6]. The authors of this study concluded that “efforts to increase minority participation in health research should focus on ensuring access to health research for all groups, rather than changing minority attitudes.” Intense research must be conducted in the areas of biology and genetics and resources must be devoted to clinical interventions, health services research, behavioral research, and community-based preventive research.

To eliminate disparities in health, we must be committed to effecting changes in policies so that universal access is a reality in this country, and that access ensures and provides quality care for all. The elements for a successful action plan include universal health insurance; a primary medical home for every citizen; proportionate representation of minority populations in health professions; bias-free interventions; nonviolent and exercise-friendly neighborhoods; nutritious food outlets; educational equality; career opportunities; parity in income and wealth; homeownership; and hope.

## Adapting the McKinlay Model to Eliminate Health Disparities

The McKinlay Model [7–9], which has been used in the context of increasing physical activity in older adults and improving child nutrition, identifies three levels for the involvement of the individual, community, and policy makers that can be targeted in the elimination of health disparities. These three levels are labeled *downstream*, *midstream*, and *upstream*.

### Downstream.

Here the focus is on the individual and his or her lifestyle or behavior. Regular physical activity, good nutrition, and compliance with immunization schedules are emphasized, as well as the importance of avoidance of toxins such as tobacco and excessive alcohol. At the downstream level we have the challenge of educating and motivating individuals toward healthy lifestyles. Changing individual behavior is never easy and it remains one of the greatest challenges in medicine and public health.

### Midstream.

The midstream focus is on communities and institutions within communities. The availability of safe streets, walking and biking trails, and safe, well-equipped parks is critical for prevention of injury and illness. Schools are urged to provide physical education in grades K–12 (primary and secondary education) and to teach and model good nutrition. The availability of supermarkets with affordable fresh fruits and vegetables is equally important. The challenge here is that many communities do not have the resources to make these changes.

### Upstream.

This is the level at which policies that support the elimination of health disparities must be made. School boards and legislators can mandate physical education in K–12 and limit vending machines at schools to healthy foods or at least a balance of foods. The United States Department of Agriculture can require schools that participate in federal food programs to provide health-enhancing foods, beginning with the breakfast program. Those same schools can be required to create programs to help children to develop lifetime habits of physical activity and good nutrition. To improve the health and future outlook of our children, we must advocate for these changes.

## Conclusion

We all have roles to play in the elimination of ethnic disparities in health. We must all care enough; we must know enough; we must do enough; and we must persist in our efforts until health disparities are eliminated in this country.
